# Application of an Inter-Species Extrapolation Method for the Prediction of Drug Interactions between Propolis and Duloxetine in Humans

**DOI:** 10.3390/ijms21051862

**Published:** 2020-03-09

**Authors:** Thi Lien Ngo, Chung-Hee Lee, Nayoung Han, Hyun-Moon Back, Su-Jin Rhee, Keumhan Noh, Hwi-Yeol Yun, Wonku Kang, Jung-Woo Chae

**Affiliations:** 1 College of Pharmacy, Chungnam National University, Daejeon 34134, Korea; ngolienvp@gmail.com (T.L.N.); choonghii@o.cnu.ac.kr (C.-H.L.); hyyun@cnu.ac.kr (H.-Y.Y.); 2 College of Pharmacy, Seoul National University, Gwanak-ro 1, Gwanakgu, Seoul 08826, Korea; hans1217@snu.ac.kr; 3 Department of Pharmaceutics, Ernest Mario School of Pharmacy, Rutgers, The State University of New Jersey, Piscataway, NJ 08854, USA; hyunmoon.back@rutgers.edu; 4 Department of Clinical Pharmacology and Therapeutics, Seoul National University College of Medicine and Hospital, Seoul 03080, Korea; rheesjin@snu.ac.kr; 5 Department of Pharmaceutical Sciences, Leslie Dan Faculty of Pharmacy, University of Toronto, Toronto, ON M5S 3M2, Canada; keumhan.noh@utoronto.ca; 6 College of Pharmacy, Chung-Ang University, Seoul 06974, Korea

**Keywords:** duloxetine, propolis, drug interaction, extrapolation, CYP1A2

## Abstract

Duloxetine (DLX) is a potent drug investigated for the treatment of depression and urinary incontinence. DLX is extensively metabolized in the liver by two P450 isozymes, CYP2D6 and CYP1A2. Propolis (PPL) is one of the popular functional foods known to have effects on activities of CYPs, including CYP1A2. Due to the high probability of using DLX and PPL simultaneously, the present study was designed to investigate the potent effect of PPL on pharmacokinetics (PKs) of DLX after co-administration in humans. A PK study was first conducted in 18 rats (*n* = 6/group), in which the plasma concentration of DLX and its major metabolite 4-hydroxy duloxetine (4-HD) with or without administration of PPL was recorded. Population PKs and potential effects of PPL were then analyzed using NONMEM software. Lastly, these results were extrapolated from rats to humans using the allometric scaling and the liver blood flow method. PPL (15,000 mg/day) exerts a statistically significant increase in DLX exposures at steady state, with a 20.2% and 24.6% increase in DLX $C_{max,ss}$ and the same 28.0% increase in DLX $AUC_{ss}$ when DLX (40 or 60 mg) was administered once or twice daily, respectively. In conclusion, safety issues are required to be attended to when individuals simultaneously use DLX and PPL at high doses, and the possibility of interactions between DLX and PPL might be noted.

## 1. Introduction

Propolis (PPL) is a resin-like material made by honeybees from the buds of poplar and cone-bearing trees and is used in beehive construction and maintenance. PPL has been used in traditional medicine for centuries worldwide [1,2]. A significant body of evidence has shown that extracts of PPL have anti-inflammatory [3], anticancer [4,5], antioxidant [6], antifungal [7], antibacterial [8], and antivirus [9] activities. These biological activities depend mainly upon the chemical composition of the constituents, which vary considerably depending on the type of plants accessible to the bees as well as the sites of collection. Currently, PPL is widely used as popular functional foods and beverages that found in many health food stores for treating various diseases. One of the most common applications of PPL is to support the immune system for treatment of cold syndromes, including upper respiratory tract infections, the common cold, and flu-like infections. Since PPL is considered to be a health supplement, not a drug, PPL dosage is generally recommended by manufacturers on the product label without any guideline from heath organizations.

Previous literature has reported that PPL has protective effects against chemical-induced liver injury [10,11,12]. For example, PPL pretreatment protected against acetaminophen-induced hepatotoxicity in mice. One of the protective mechanisms was that PPL inhibited the formation of *N*-acetyl-*p*-benzoquinoneimine (an active metabolite of acetaminophen), which was produced by cytochrome P450 (CYP) enzymes [10]. Changes in the activity of CYP450 enzymes is one of the reasons for interactions between supplements and drugs that may cause adverse events. Therefore, effects of PPL and its major ingredients on CYP450 activities has been characterized recently [13,14,15,16]. For instance, Naramoto et al. has reported that ethanol extraction of Brazilian Green PPL inhibited enzymatic activities of all the tested CYP enzymes (CYP1A2, 2C9, 2C19, 2D6, and 3A4) *in vitro* in a concentration-dependent manner [13]. The inhibition effects of PPL on CYP1A2, 2E1, and 2C19 activities *in vitro* and *in vivo* have also been reported in our recently study [14]. Interestingly, none of published studies had evaluated the interactions of PPL with clinical drugs in humans, although PPL has been used widely as a health supplement.

Duloxetine (DLX), (+)-(S)-N-methyl-γ-(1-naphthyloxy)-2-thiophenrpropylamine, administered as duloxetine hydrochloride, is a potent and selective serotonin and norepinephrine reuptake inhibitor. This drug is used clinically in many countries for the treatment of disorders related to norepinephrine and serotonin, including major depressive disorder, diabetic peripheral neuropathic pain, and generalized anxiety disorder [17,18]. The drug is rapidly and extensively metabolized by CYP1A2, and to a lesser degree, by CYP2D6, to form multiple oxidative metabolites, which are then conjugated before being excreted in the urine [19]. Correspondingly, the glucuronide conjugate of 4-hydroxy duloxetine (4-HD) and the sulfate conjugate of 5-hydroxy-6-methoxy duloxetine are the two major metabolites of DLX in plasma [19]. A clinical study by Lobo et al. [20] reported that following a single oral DLX dose, the co-administration of fluvoxamine (a strong CYP1A2 inhibitor) resulted in clinically important increases in the DLX $AUC_{0-\infty }$(460%) and $C_{max}\;$(141%). To explain these significant increases, the authors suggested that fluvoxamine increased the amount of DLX absorbed into systemic circulation by decreasing the extent of first-pass metabolism and, additionally, decreased systemic clearance of DLX by inhibiting CYP1A2-mediated metabolism.

Since only DLX has pharmacological effects, while all its circulating metabolites are inactive, any interaction resulting in a variation in DLX metabolism may alter its therapeutic effects, safety, and/or adverse events [17]. Nowadays, many people, especially elderly people, usually take drugs and supplementary simultaneously. As PPL is widely used for addressing various diseases, there is a high probability that DLX and PPL are administered at the same time. This study was conducted to investigate potential effects of PPL on the pharmacokinetic (PK) profile of DLX when they are co-administered in humans, using population PK model-based approach [21,22]. First, a PK study for drug interactions between DLX and PPL was conducted in rats. A population PK model was subsequently developed using nonlinear mixed-effects modelling (NONMEM) software to describe the PK profiles of DLX and its major metabolite 4-HD in rats. The results were then extrapolated from rats to humans by applying the simple allometric scaling method [23,24,25,26] and the liver blood flow (LBF) method [27,28].

## 2. Results

### 2.1. PK Data of DLX and 4-HD in Rats

Plasma concentration of DLX and 4-HD after a single oral dose of 40 mg/kg DLX or a single oral dose of 40 mg/kg DLX with 500 or 1500 mg/kg PPL in rats were determined using a high-performance liquid chromatography-tandem mass spectrometer (HPLC-MS/MS) method. The calibration curves established by plotting the peak area ratio vs. concentration were linear in the range of 5–1000 ng/mL for both of these two analytes (R^2^ > 0.99). During the method validation, all the test results met the required criteria according to the U.S Food and Drug Administration (FDA) guidelines for bioanalytical method validation [29]. Individual and mean of population of plasma concentration-time curves of DLX and 4-HD are illustrated in Supplementary Materials Figure S1.

### 2.2. Population PK Model for DLX and 4-HD in Rats

The final population PK model used to describe the PKs of DLX and its metabolite 4-HD with characterizing the metabolism of DLX converted to 4-HD was chosen after testing various models based on the objective function value (OFV) changes and goodness of fit (GOF) plots. A summary of building steps for covariate models resulting in statistical significance in the OFV during the model development is listed in Table 1. As demonstrated, PPL showed a significant inhibitory effect (*p* < 0.000) on the fraction of 4-HD converted from DLX ($F_{pm}$) at the depot, as well as the conversion of DLX to 4-HD ($CL_{pm}$) at the systemic circulation.

($F_{pm}$) and systemic ($CL_{pm}$) metabolisms.

**Figure 1:**
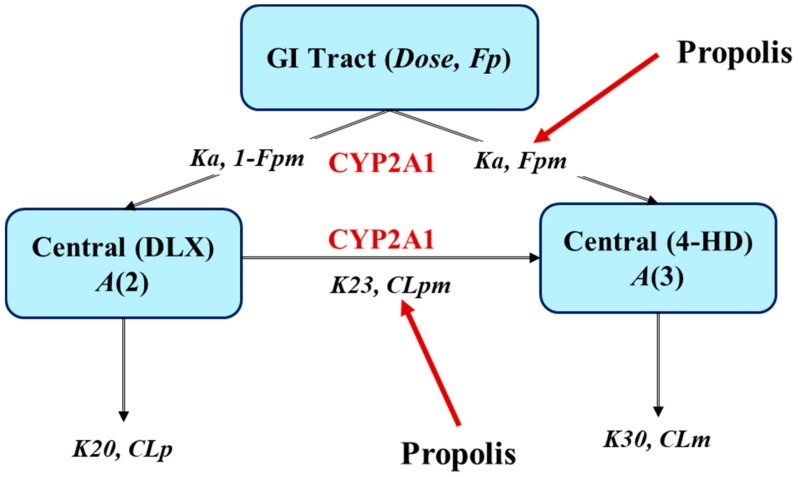
Schematic representation of the base population pharmacokinetic (PK) model for DLX and its metabolite, 4-HD, and strategies to investigate the effect of PPL on the PKs of DLX after a single oral dose of 40 mg/kg DLX in rats. Notations are described in the main text. Pre-systemic ($F_{pm}$) and systemic ($CL_{pm}$) metabolisms.

**Figure 2:**
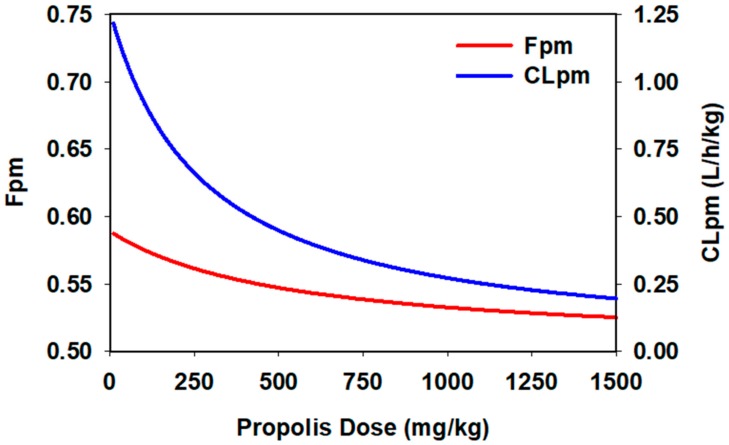
Predicted conversion fractions of DLX to 4-HD via the pre-systemic ($F_{pm}$) and systemic ($CL_{pm}$) metabolisms according to the different administered doses of PPL.

PKs of DLX and 4-HD were well described by a one-compartment disposition model, with first-order elimination and first-order absorption kinetics. The metabolism of DLX converted to 4-HD was described by two metabolism pathways, including the first-pass effect and the systemic metabolism (Figure 1). The covariate effects of PPL on PK of DLX were modeled using a Michaelis Menten equation, as described in the following equations (Equations (1) and (2)) and illustrated in Figure 2.

(1) $$CLpm=1.26\times \left(1-1\times \frac{Dose_{PPL}}{Dose_{PPL}\;+\;276}\right)$$

(2) $$Fpm=0.589\times \left(1-0.147\times \frac{Dose_{PPL}}{Dose_{PPL}\;+\;538}\right)$$

By using the final model, only clearance of DLX via all elimination routes except the conversion to 4-HD via the systemic metabolism from the body ($CL_{p}$) need to be modeled with inter-individual variation effect. Estimates of fixed effects, intra- and inter-individual random effects, and their relative standard errors (% RSE) for the estimations are listed in Table 1. As shown in Table 2, the PK parameters of DLX and 4-HD in rats were generally well-estimated, with their % RSE in the range of 8.10%–85.7%, except *IC50_*$F_{pm}$ (358%). The IIV estimate for $CL_{p}$ was 7.9% with an RSE of 70%. Accordingly, a decrease by 149% (from 149% to 0%) in IIV of $CL_{pm}$ and a decrease by 10.5% (from 18.4% to 7.9%) in IIV of $CL_{p}$ were observed in comparison to the base model. The proportional residual error was 19.9% (for DLX) and 24.0% (for 4-HD).

**Figure 3:**
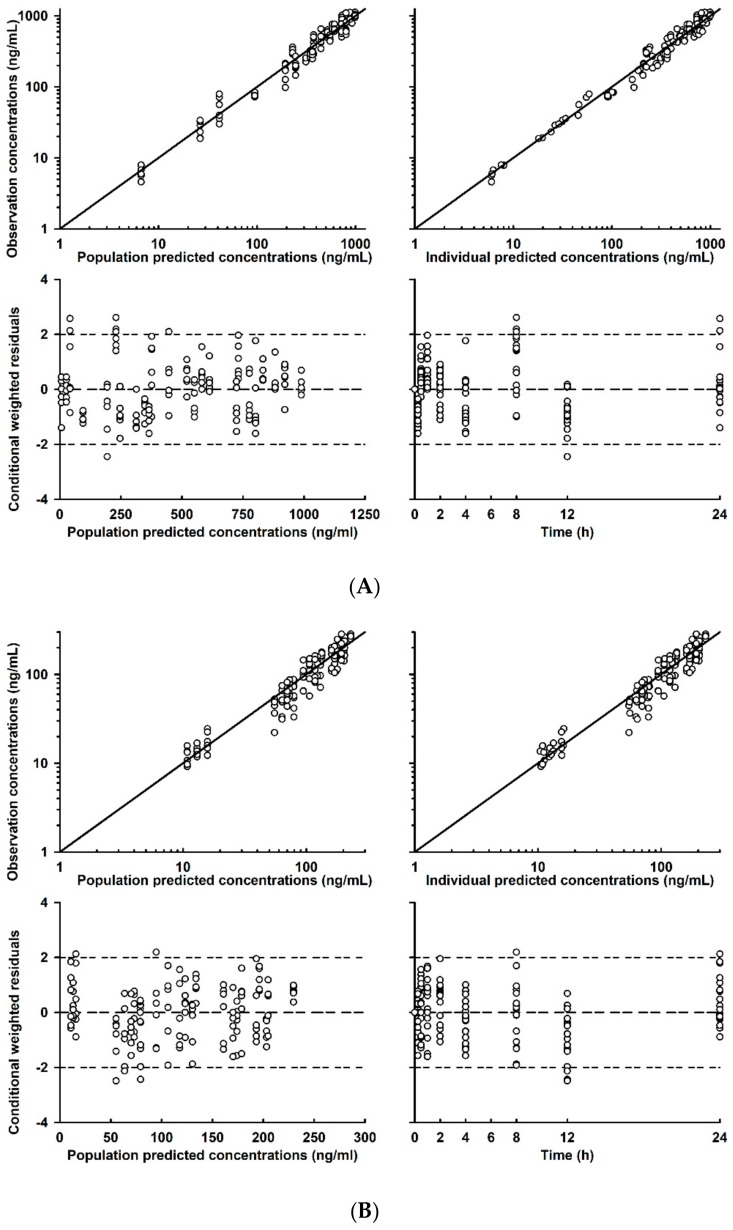
Goodness of fit plots for the final model for predictions of the PK profiles of (**A**) DLX and (**B**) 4-HD in rats.

The basic GOF plots of the final model of the plasma concentration-time profiles of DLX and 4-HD are shown in Figure 3, suggesting that the individual prediction concentrations were reasonably well fitted by the final covariate model. The population and individual post-hoc predictions were distributed around the line of identity with no systematic bias. Moreover, conditional weighted residuals for the population predicted plasma concentrations were generally distributed around zero and were symmetric.

### 2.3. Population PK Model Evaluation

**Figure 4:**
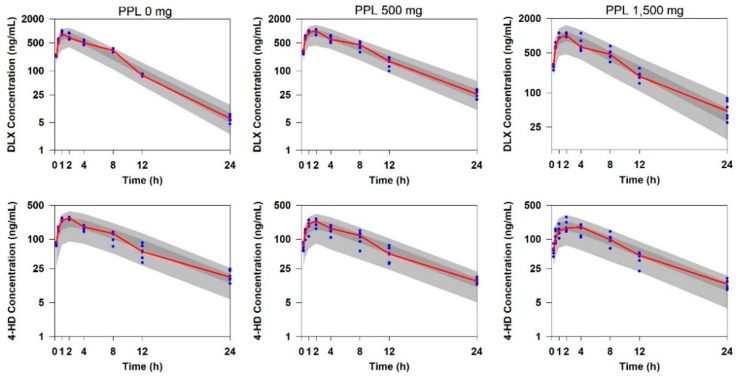
Visual predictive check plots for the final model for predictions of the PK profiles of (upper) DLX and (lower) 4-HD in rats. Open circles: observed concentrations, solid red line: median of the observed concentrations, dark grey area: 95% confidence interval (2.5^th^–97.5^th^ percentiles) of the median predicted concentrations, and light grey area: 90% prediction intervals (5.0^th^ to 95.0^th^ percentiles) of the predicted concentrations.

Evaluation and selection of the models were based on graphical analysis (visual predictive check, VPC plots) as well as statistical methods (bootstrap analysis). The predicted concentrations from 1000 simulated datasets using the final model for DLX and 4-HD after a single oral dose of 40 mg DLX and a variable dose of PPL in rats are shown in Figure 4. As illustrated in Figure 4, all of the observed concentrations were within the 5^th^–95^th^ percentiles of the simulated concentrations. In addition, the majority of the observed median values were within the 95% confidence interval of the predicted median values obtained by the simulation. Results of the bootstrap analysis are listed in Table 2, indicating the parameter estimates of the final model were in the 2.5^th^–97.5^th^ percentiles and were close to the medians estimated from bootstrap replicates. This result demonstrated that the parameter estimates of the final model were not statistically different from the parameters estimated by the bootstrap process.

### 2.4. Extrapolation of PKs of DLX and 4-HD in Humans

PK parameters of DLX and 4-HD in humans extrapolated from rats and from previous studies, including absorption rate constant $K_{a}$, elimination rate constant $K_{e}$, apparent oral clearance ($CL_{pm}/F_{p}$ and $CL_{p}/F_{p}$), volume of distribution ($\;V_{p}/F_{p}$) of DLX, where $F_{p}$ is bioavailability of DLX after the first-pass effect, and apparent clearance $CL_{m}/F_{m}$ and apparent volume of distribution $\;V_{m}/F_{m}$ of 4-HD, where $F_{m}$ is bioavailability of 4-HD, are listed in Table 3. Other PK parameters, including the effects of PPL dose on the PKs of DLX, as well as the IIV and RSE of all PK parameters in humans, were assumed to be the same as in the rats. The predicted total clearance ($CL_{DLX}/F_{p}$) and volume of distribution ($V_{p}/F_{p}$*)* of DLX was 55.9 L/h and 1022 L, respectively. Following conversion, the predicted apparent total clearance ($CL_{DLX}/F_{DLX}$) and volume of distribution ($V_{DLX}/F_{DLX}$) was 136 L/h and 2487 L, respectively, where $F_{DLX}$ is the total bioavailability of DLX. In other words, the fraction of drug that reaches the systemic circulation from the DLX dose is calculated following the Equation (3) below:

(3) $$F_{DLX}=\left(1-F_{pm}\right)\times F_{p}$$

**Figure 5:**
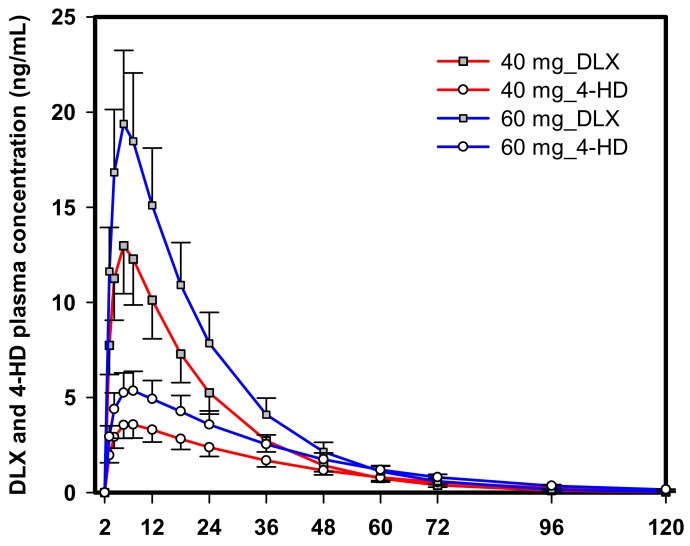
Extrapolated concentration-time curve profiles of DLX and 4-HD after a single oral administration of 40 mg or 60 mg in humans. Data are presented as means (solid square, DLX and open circles, 4-DH) and standard deviations (error bars).

The predicted PK profile of DLX and 4-HD following a single oral administration of DLX at the dose of 40 or 60 mg in humans is illustrated in Figure 5. The maximum concentration ($C_{max}$) and area under the concentration-time curves (*AUC*) from zero to last time ($AUC_{0-t}$) of DLX are listed in Table 4.

### 2.5. Extrapolation of Effect of PPL on PKs of DLX in Humans

**Figure 6:**
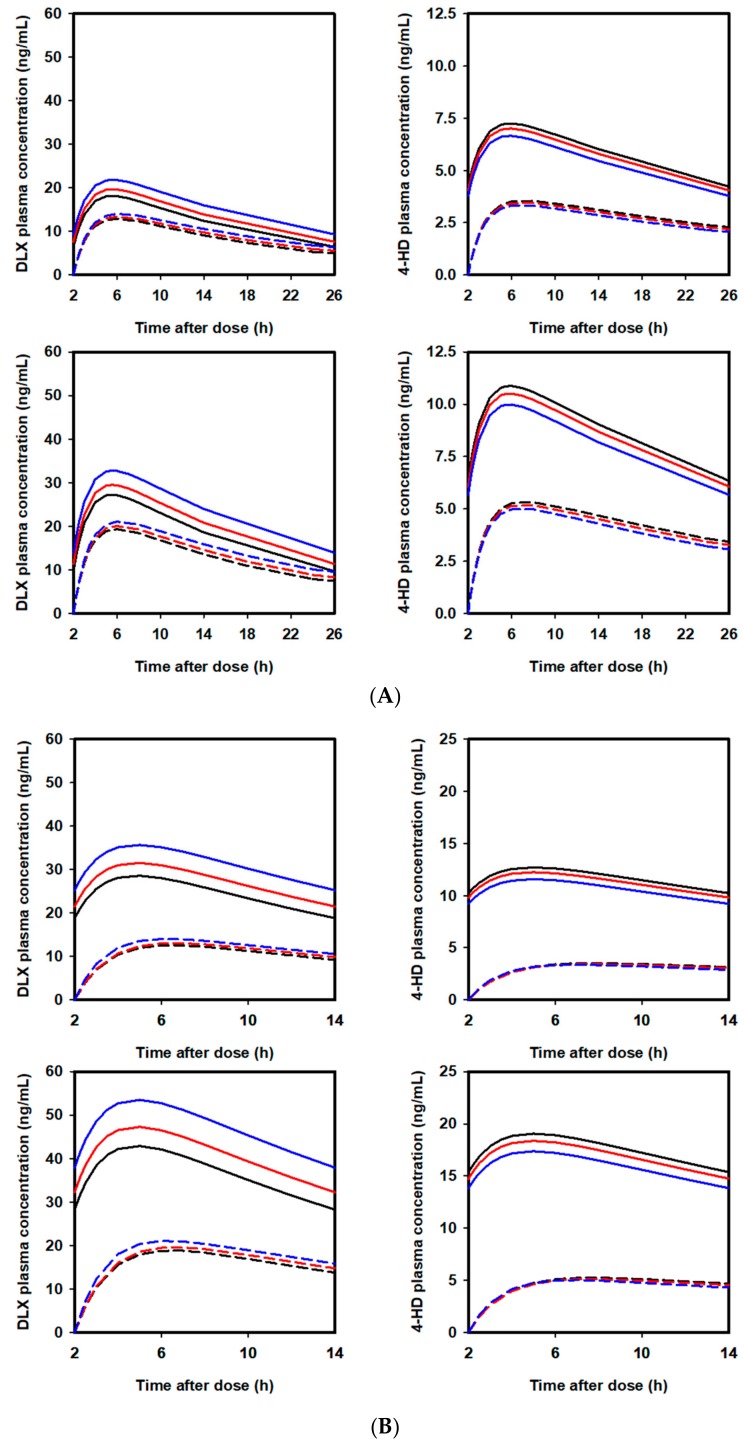
(**A**) Extrapolated concentration-time curve profiles of DLX and 4-HD at the first dosing interval (dashed line) and at the steady-state condition (solid line) following multiple DLX doses given once daily according to difference doses of PPL in humans: (black) PPL 0 mg, (red) PPL 5000 mg, and (blue) PPL 15,000 mg. (Upper) 40 mg and (lower) 60 mg; (**B**) Extrapolated concentration-time curve profiles of DLX and 4-HD at the first dosing interval (dashed line) and at the steady-state condition (solid line) following multiple DLX doses given twice daily according to difference doses of PPL in humans: (black) PPL 0 mg, (red) PPL 5000 mg, and (blue) PPL 15,000 mg. (Upper) 40 mg and (lower) 60 mg.

PK profiles of DLX and 4-HD after administration of multiple DLX 40 or 60 mg doses given once or twice daily co-administered with difference PPL doses (0, 5000, or 15,000 mg/day) in the first day and at the steady state condition are illustrated in Figure 6. The respective PK parameters of DLX are listed in Table 4. As shown in Table 4, DLX *t_1/2_* increased by 1.1 h (from 12.7 to 13.8 h) and 2.6 h (from 12.7 to 15.3 h) when the drug was co-administered with PPL at doses of 5000 or 15,000 mg/day, respectively, comparing to when the drug was administered alone. Correspondingly, when DLX was administered once daily, the presence of PPL resulted in an increase by 11.6% and 28.0% in AUC during one dosing interval ($AUC_{SS}$) and an increase by 8.35% and 20.2% in $C_{max}$ ($C_{max,SS}$) at the steady state. Additionally, when DLX was administered twice daily, the presence of PPL resulted in an increase by 11.6% and 28.0% in $AUC_{SS}$ and an increase by 10.2% and 24.6% in $C_{max,SS}$ at the steady state, respectively.

## 3. Discussions

Drug interactions are one of the most common causes of medical adverse events. Therefore, evaluating potential drug interaction is an integral part of drug development. It is also important for making dosing recommendations for co-administered drugs. PK drug interactions may occur if a drug, a supplement, or food affects any one of the following four processes of another drug: absorption, distribution, metabolism, or elimination (ADME) [21,30,31].

PPL has been found to inhibit more than 50% activities of many enzymatic CYPs in a concentration-dependent manner, including CYP1A2. Changes in activity of CYP enzymes is thought to be a factor in interactions between supplements and drugs that may cause adverse events. While DLX has been reported to be a substrate of CYP450, the drug is rapidly and extensively metabolized by CYP1A2 and, to a lesser degree, metabolized by CYP2D6 to form multiple oxidative metabolites [19]. Consequently, there are probability of interactions between DLX and PPL through CYP1A2-mediated inhibition of PPL when they are co-administered in humans.

Approaches to predict human PK profiles from *in vitro* and/or PK profiles in preclinical species, utilizing both physiological and empirical approaches, are highly desirable [32,33,34,35,36,37,38,39], because this can drastically reduce the time and expense of drug development and dose regiment. Allometric scaling, an empirical method, has been intensively studied and widely applied to predict many important PK parameters, including *CL*, *Vd*, and *t_1/2_*, due to its simplicity [23,24,25,26]. However, allometric scaling has limitations, especially since it assumes that there are anatomical, physiological, and biochemical similarities among animals. The method may not be suitable for drugs that exhibit species-specific differences; for instance, drugs with high protein binding properties, significant biliary excretion, extensive active renal secretion, active metabolism, or show species-specific binding [40,41,42]. DLX is one of these cases. The drug is primarily cleared by hepatic metabolism via the activity of CYP1A2 and CYP2D6. Therefore, another method instead of a simple allometric scaling method, which can account for the difference in hepatic elimination functions between humans and rats, is required. As a result, an LBF method was selected, because it assumed that a drug clearance is proportional to the LBF of the subject; therefore, it can account for the difference in the liver functions of inter-species. By which methods, the mean apparent total clearance ($CL_{DLX}/F_{DLX}$), volume of distribution ($V_{DLX}/F_{DLX}$), and terminal elimination half-life *t_1/2_* of DLX in humans was estimated to be 136 L/h, 2487 L, and 12.7 h, respectively. They are all in agreement with published literature [17,18,19,20,43,44,45,46,47]. For instance, according to the product information of Cymbalta (duloxetine hydrochloride) by the FDA, the volume of distribution of DLX in humans was 1640 L [17]. In the EMA product information, the apparent plasma clearance of DLX ranges from 33 to 261 L/h (mean 101 L/h) after oral administration of DLX [18].

Up until now, there have been no official criteria for extrapolated data from animals to humans. However, the criteria that the prediction of PK parameters within the range of one-half and twice (also known as two-fold dimensions) of the observed data are determined to be acceptable predictions has been suggested and applied in many published studies [28,48,49,50]. Therefore, we also applied the two-fold dimension criteria to assess the success/failure of our predicted data. Accordingly, the present study succeeded in predicting $CL_{DLX}/F_{DLX}$ and $V_{DLX}/F_{DLX}$ parameters of DLX in humans.

The absorption rate constant of DLX in humans was predicted based on the relationship between $K_{a},\;K_{e},$ and time to reach $C_{max}\;$($T_{max}$), as described in Equation (4):

(4) $$T_{max}=\frac{\ln Ka-\ln Ke}{Ka-Ke}$$

Given that DLX is acid labile, it therefore must be formulated with enteric-coated pellets to protect DLX from degradation in the acidic stomach environment by preventing DLX dissolution in the acidic stomach but allowing immediate release and rapid absorption at the small intestine. Consequently, after oral administration, there is a median 2-h lag time ($T_{lag}$) until absorption begins [17]. Therefore, the $T_{max}$ in Equation (4) was calculated as the time from when the absorption begins until the time when the $C_{max}$ reaches, Equation (5) as follows:

(5) $$T_{max}=T_{max,post\;dose}-T_{lag}$$

As a result, $K_{a}$ was predicted to be 0.697 1/h, which also is in agreement with the experimental estimate in humans by Sharma et al., where Ka was around 0.6 1/h [51].

By applying the above-extrapolated PK parameters from rats to humans, the mean $C_{max}$ and $AUC_{0-t}$ of DLX after a single oral administration of 60 mg DLX were predicted to be 19.4 ng/mL and 441 h*ng/mL, respectively, which are reasonably similar to values reported in previous literature [20].

In conclusion, the PKs of DLX were successfully extrapolated from rats to humans by applying a method that accounted for both the pre-systemic and systemic metabolism of DLX to 4-HD. The extrapolation method developed here was further applied to investigate the potential effects of PPL, a CYP1A2 inhibitor, to the PKs of DLX when they are co-administered in humans.

Since PPL is considered to be only a health supplement, not a drug, the PPL dosage is generally recommended by manufacturers without any guidance from health organizations. PPL is designed in a capsule formulation, and one capsule contains up to 500 mg dry extract that is equivalent to up to 5000 mg fresh natural PPL. The recommended dose for this product is one to three capsules daily [52,53,54,55]. The recommended dosing regimen of DLX is 60 mg daily (given as 60 mg once daily or 30 mg twice daily) for major depressive disorder, diabetic peripheral neuropathic pain, and generalized anxiety disorder [17]. For stress urinary incontinence, the recommended dose is 40 mg twice daily. Based on the response of patients to treatment, the dose may be adjusted up to 120 mg daily (given as 60 mg twice daily). To mimic the therapeutic dosage of DLX and PPL, we extrapolated our data at different scenarios, including DLX 40 or 60 mg given once or twice daily and PPL 5000 mg/day (the medium dose) or 15,000 mg/day (the maximum dose). Due to a terminal elimination half-life of 12 h, which allows steady-state conditions to be achieved after three days, PKs of DLX and 4-HD were extracted after seven days post-dose to confirm the steady-state condition.

As a result, PPL (15,000 mg/day) exerts a statistically significant increase in DLX exposures at steady state, with a 20.2% and 24.6% increase in DLX $C_{max,ss}$ and the same 28.0% increase in DLX $AUC_{ss}$ when DLX was administered once or twice daily, respectively. However, a previous study has reported that despite higher DLX plasma concentrations during co-administration with fluvoxamine (138% increase in $C_{max}$ and 460% increase in $AUC_{0-\infty }$), adverse events were not observed more often than when DLX was used alone [20]. In addition, according to the FDA and European Medicines Agency (EMA), the safety of DLX up to 120 mg has been adequately established for the approved indications [17]. These results suggested that when DLX at the dose less than 120 mg was administered, the increased DLX exposure due to co-administration with PPL (even at the highest PPL dose) are still within the safety range, and it may not cause clinically important safety concerns. However, at the highest DLX dose (60 mg, twice daily; equivalent to 120 mg/day), safety issues need to be concerned, because there has been no safety test performed on doses greater than 120 mg/day. In addition, due to these changes in PKs of DLX, in all cases, we suggest that the probability of interaction between DLX and PPL should to be noticed by manufacturers and health organizations.

The present study had some limitations. The inhibitory CYP1A2 effect of PPL on PKs of DLX was conducted in rats, followed by extrapolation into healthy human subjects directly. The extrapolation was conducted with the assumption that there are anatomical, physiological, and biochemical similarities among rats and humans. However, animal populations may not exactly represent human populations in these conditions. In addition, healthy subjects also may not well represent the patient populations. Therefore, to make future decisions about using a treatment combination of DLX and PPL, for instance, whether the combination should be avoided or patient safety should be monitored during co-administration in humans, future clinical studies should be conducted in diverse human populations, especially patients with liver or renal failures and patients treated with DLX. Importantly, as we mentioned above, the biological activities in PPL depend mainly upon the chemical composition of the constituents, which vary considerably depending on the type of plants accessible to the bees as well as the sites of collection. As the results of the drug interaction between PPL and duloxetine could be different depending on the major ingredients and its respective contents of PPL, its interpretation should be paid attention to.

## 4. Material and Methods

### 4.1. Quantitative Determination of Major Ingredients in Propolis Extract

PPL capsules were purchased from Natural Immix Health Ltd. (Port Coquitlam, BC, Canada) and Country Life Ltd. (Hauppauge, NY, USA) and pooled at the ratio 1:1 (*w*/*w*). The PPL capsules contained only PPL extract from beehives of the honey bee without additives. PPL capsules were dissolved to 200 μg/mL in 0.1 M phosphate buffer (pH 7.4) containing 0.5% dimethyl sulfoxide (DMSO). Major ingredients in the PPL extract were then determined by a HPLC–MS/MS system as described in our previous report [14]. The quantities of major ingredients are shown in Table 5.

### 4.2. PK Study Design in Rats and Data Analysis

DLX and 4-HD were purchased from Waterstone Technology (Carmel, IN, USA) and Santa Cruz Biotechnology (Santa Cruz, CA, USA), respectively. Carbamazepine, used as an internal standard (IS) for analyzing DLX and 4-HD, was purchased from Sigma Chemical (St Louis, MO, USA).

The present study was approved by the Animal Ethics Committee of Chungnam National University (no. CNU-00721). It was conducted in 18 SD rats divided into 3 groups (G1, G2, and G3; *n* = 6 rats/group). Each was orally administered the same DLX dose (40 mg/kg) and a different PPL dose (G1, 0 mg/kg; G2, 500 mg/kg; and G3, 1500 mg/kg). PPL doses in the present study were the equivalent amounts of natural PPL. Blood samples (0.3 mL) were collected in Vacutainer^®^ tubes (Becton Dickinson and Company, Franklin Lakes, NJ, USA) at pre-dose (0 h) and at various predetermined time-points (0.25, 0.5, 1, 2, 4, 8, 12, and 24 h) post-dose. Samples were centrifuged; the plasma was then transferred to polyethylene tubes and stored at −70 °C until analysis.

DLX and 4-HD were extracted from the plasma by a liquid-liquid extraction method, followed by analysis with a validated HPLC–MS/MS method as described in our previous report [56]. In detail, DLX and 4-HD were ionized in the positive electrospray ionization mode and detected through multiple reaction monitoring transitions at 298.00→44.00 and 314.13→154.10 *m*/*z*, respectively. Calibration curves were linear over a concentration range of 5–1000 ng/mL for both these two analytes (R^2^ > 0.99). Separation was performed using an Atlantis C18 (50 mm × 4.6 mm, 3 mm; Waters, Dublin, Ireland) column operated at 40 ± 5 °C. The mobile phase was a mixture of methanol with 5 nM ammonium acetate at a volume ratio of 60:40 and a flow rate of 0.3 mL/min.

### 4.3. Rat Population PK Model Development

PK analysis was conducted using a software package for nonlinear mixed-effects modeling (NONMEM version 7.3.0, ICON Development Solutions, Hanover, MD, USA) [57]. The PK model was estimated using the first-order conditional estimation (FOCE) method with interaction. First, a base model was developed without characterization of the effect of PPL on the PKs of DLX. The absorption and elimination were assumed to follow first-order kinetics. The lognormal distribution was used to model the IIV in the PK parameters, as described by Equation (6).

*P_i_* = *P_TV_* × exp(*ƞi*) (6)

where *P_i_* is the parameter value for the *i*th individual, *P_TV_* is the typical population parameter, and *ƞ_i_* (IIV) is the difference between *P_i_* and *P_TV_* that is assumed to be normally distributed with a mean of zero and a variance of *ω_ƞ_^2^*. A proportional error model was applied to describe the residual random error (*ε*) that contains contributions from IIV, assay error, and model misspecification, as expressed by Equation (7):

*C_ij_ = C*_pred,*ij*_ × (1 + *ε*_pro*,ij*_) (7)

where C*_ij_* and *C*_pred,*ij*_ present the *j*th observed and predicted concentrations for the *i*th individual, respectively; *ε*_pro*,ij*_ is the proportional component that has normally distributed residual random effects with a mean of zero and a variance of *σ*^2^_pro_.

The proposed disposition compartment model for DLX and its metabolite, 4-HD, is schematically presented in Figure 1. This model suggests two pathways of 4-HD formation from DLX: (1) pre-systemic metabolism and (2) systemic metabolism. Correspondingly, the estimated population PK parameters for 4-HD were a fraction of the metabolite converted from DLX at the depot ($F_{pm}$), which was exerted by the first-pass liver metabolism, and the rate of the metabolite converted from DLX circulating in the plasma ($K_{pm}$), which exerted by systemic metabolism.

The estimated population PK parameters for DLX were apparent oral clearance ($CL_{pm}/F_{p}$ and $CL_{p}/F_{p}$), volume of distribution ($V_{p}/F_{p}$), and the absorption rate constant $K_{a}$. Where $CL_{pm}$ is the clearance of DLX via conversion into 4-HD; $CL_{p}$ is the clearance of DLX via all routes except for conversion into 4-HD; $F_{p}$ is bioavailability of DLX after the first-pass effect. In other words, $F_{p}$ is the fraction of the drug that reaches the systemic circulation in relation to the amount of DLX ($Amount_{DLX}$) that “survives” after passing the liver from the depot. The $Amount_{DLX}$ is calculated following the Equation (8) below:

(8) $$Amount_{DLX}=Dose_{DLX}\times \left(1-F_{pm}\right)$$

Second, a covariate model to describe the possible effect of PPL on the PKs of DLX through its interaction with CYP1A2 was developed based on the base model. Covariate model building was accomplished by mixed stepwise forward addition (*p* < 0.05) and stepwise backward elimination (*p* < 0.01) based on change in the OFV using a likelihood ratio test within NONMEM, as well as reductions in IIV of PK parameters, GOF plots, and precision of estimates. Effects of PPL on the metabolism of DLX were modeled using a Michaelis Menten equation. For example, the effects of PPL on $CL_{pm}$ is described by the Equation (9) as follows:

(9) $$CL_{pm}=TVCL_{pm}\times \left(1-Emax_{CLpm}\times \frac{Dose_{PPL}}{Dose_{PPL}\;+\;IC50_{CLpm}}\right)$$

where $TVCL_{pm}$ is the typical value of $CL_{pm}$ in the population without co-administration with PPL, $Emax_{CLpm}$ is the maximum inhibition effect of PPL on $CL_{pm}$, and $IC50_{CLpm}$ is the co-administered dose (mg/kg) of PPL at which PPL has 50% of the maximum inhibition effect.

### 4.4. Rat Population PK Model Evaluation

Evaluation and selection of the models were based on graphical analysis as well as statistical methods. A nonparametric bootstrap analysis and a VPC were performed by simulating 1000 replicates each group using NONMEM. The final model assumed the same study design with same parameter estimates and their variances. Concentrations predicted from the VPC simulations were plotted against time for a 95% CI of the median, 5^th^, and 95^th^ percentiles and overlaid with observed data. One thousand bootstrap replications were performed in PsN on the final model to obtain the median and 95% nonparametric CI (the 2.5^th^ and 97.5^th^ percentiles) for all parameters to assess parameter uncertainty.

### 4.5. Extrapolating Population PK of DLX in Humans

Assuming that an adult human has an average body weight of 70 kg, *Vd* of DLX and 4-HD in humans was extrapolated from the PK parameters in rats using a simple inter-species allometric scaling method [23,24,25,26], which assumes the similarities among animals can be generalized and expressed mathematically by an allometric relationship, as presented in Equation (10):

(10) $$Y\;=a\times W^{b}$$

where *Y* is the parameter of interest, *W* is the average body weight of a species, and *a* and *b* are the allometric coefficient and exponent of the equation, respectively. In the case of *Vd*, a fixed value of 1.0 was used for the exponent in the equation [23,24,25,26]. Accordingly, the *Vd* in humans was predicted from the rat PK parameter, as presented in Equation (11):

(11) $$V_{human}=V_{rat}\times {\left(\frac{BW_{human}}{BW_{rat}}\right)}^{1.00}$$

Clearance (*CL*) in humans was extrapolated from rats following a method described by Ward and Smith [27], wherein *CL* is considered to be proportional to *LBF*. Accordingly, human *CL* was predicted following the Equation (12) below:

(12) $$CL_{human}=CL_{rat}\times \left(\frac{LBF_{human}}{LBF_{rat}}\right)$$

where *LBF* values for rats and humans are 85 and 21 mL/min per kg of body weight, respectively [27].

The absorption rate constant was predicted based on an equation that describes the relationship between $K_{a}$, elimination rate constant $K_{e}$, and time to reach $C_{max}$ ($T_{\max})$, as expressed by Equation (13):

(13) $$T_{\max}=\frac{\ln Ka-\ln Ke}{Ka-Ke}$$

where $\;T_{\max}$ was extracted from literature reports for PKs of DLX in humans and $K_{e}$ was calculated following the Equation (14) below:

(14) $$Ke_{human\;}=\frac{CL_{human}}{V_{human}}=\frac{CLpm+CLp}{Vp}$$

To determine whether the extrapolation from rats to humans was successful or not, the extrapolated PK parameters were compared directly with the corresponding values estimated experimentally in humans. The extrapolation was assessed to be successful when the extrapolated human PK parameters lay within two-fold dimensions of the experimental values. Otherwise, it was assessed to be unsuccessful [28,48,49,50].

### 4.6. Simulation of PPL Effects on PKs of DLX in Humans

A potential effect of co-administered PPL on PKs of DLX in humans was performed by simulating 1000 replicates using the developed population PK model in rats with DLX and 4-HD. PK parameters in humans were obtained from extrapolation, as described in Section 2.4. The IIV between subjects in the human population was assumed to be the same as those in the rat population. In addition, the effects of PPL on the PKs of DLX were assumed to be constant across species.

Six scenarios for DLX and PPL co-administrations were simulated. For DLX, the drug was administered as follows: (1) 40 or 60 mg once daily and (2) 40 or 60 mg twice daily. For PPL, the supplement was co-administered with DLX at a total dose of (1) 0 mg, (2) 5000 mg, or (3) 15,000 mg of PPL per day. The potential effects of PPL on the PKs of DLX were assessed by comparing *AUC* and $C_{max}$ of DLX with co-administration (PPL = 5000 or 15,000 mg) and without co-administration (PPL = 0) of PPL.

## 5. Conclusions

After a single oral dose of DLX 40 mg/kg in rats, PKs of DLX and its metabolite 4-HD were modeled well by a one-compartment disposition model with first-order absorption and first-order elimination. While the metabolism from DLX to 4-HD was described by both pre-systemic (first-pass effect) and systemic metabolic pathways, a Michaelis-Menten equation was used to model the CYP1A2 inhibition kinetics of PPL on the metabolism of DLX converted to 4-HD. The PK profiles of DLX, as well as the interaction of PPL with the PKs of DLX in humans, were successfully extrapolated from the corresponding PK parameters in rats by applying an allometric scaling method (for predicting volume of distribution), an LBF method (for predicting clearance), and other PK parameters in humans extracted from experimental literature, with the final developed population model in rats. In conclusion, at the DLX doses less than 120 mg, the presence of PPL exerts a statistically significant increase in DLX exposures but does not seem to exert clinically significant changes in the PKs of DLX, even at the maximum dose of PPL (15,000 mg/day). At the maximum therapeutic dose of DLX (60 mg twice daily), safety issues are required to be attended to, and the possibility of interaction between DLX and PPL might be noted. Future studies further investigating these findings should be conducted in diverse human populations to provide a better representation.

## Figures and Tables

**Table 1:** Summary of covariate model building steps.

Model No.	Model Description	df.	Change in OFV	Compared with	Significance (*p*-Value)
1	Base model	−	−	−	−
2	Model 1 with PPL as covariate for *Fpm*	1	−38.4	Model 1	<0.000
3	Model 1 with PPL as covariate for *CLpm*	2	−28.7	Model 1	<0.000
4 ^a^	Model 1 with PPL as covariate for *Fpm* and *CLp*	3	−47.8	Model 1	<0.000
4 ^a^	Model 1 with PPL as covariate for *Fpm* and *CLp*	2	−10.6	Model 2	<0.005

OFV, objective function value; df., degrees of freedom; and ^a^ final model. Pre-systemic (*F_pm_*) and systemic ($CL_{pm}$) metabolisms.

**Table 2:** Parameter estimates from the final model and results of bootstrap validation for DLX and 4-HD after a single oral administration of DLX at dose of 40 mg without or with co-administration with PPL 500 or 1500 mg/kg in rats.

Parameters	Unit	Estimates	RSE (%)	Shrinkage (%)	Bootstrap Replicates (*n* = 1000)			
					Median	95% CI		
$K_{a}$	1/h	1.35	8.10		1.35	1.24	−	1.45
*Emax_* $F_{pm}$		0.147	85.7		0.198	0.0435	−	0.844
*IC50_* $F_{pm}$	mg/kg	538	358		806	45.7	−	5478
$F_{pm}$		0.589	15.9		0.574	0.451	−	0.746
$CL_{p}$	L/h/kg	1.97	22.6		2.10	1.08	−	2.80
$V_{p}$	L/kg	14.6	26.4		15.1	9.01	−	19.6
*Emax_* $CL_{pm}$		1.00			1.00	1.00	−	1.00
*IC50_* $CL_{pm}$	mg/kg	276	77.2		270	60.1	−	596
$CL_{pm}/F_{p}$	L/h/kg	1.26	30.7		1.24	0.770	−	1.77
$CL_{m}/F_{m}$	L/h/kg	12.3	13.6		11.9	9.45	−	16.0
$V_{m}/F_{m}$	L/kg	84.2	21.6		81.3	64.3	−	110
Inter-individual variability (IIV, %)								
IIV $CL_{p}$		7.90	70.0	14.0	7.10	3.21	−	10.2
Residual variability (%)								
*Prop_p*		19.9	13.4		19.8	18.0	−	21.9
*Prop_m*		24.0	10.3		23.5	20.8	−	26.2

RSE, relative standard error; IIV, inter-individual variation; CI, confidence interval (the 2.5^th^ and 97.5^th^ percentiles); *Prop_p* and *Prop_m* are the proportional residual errors for DLX and 4-HD, respectively; $F_{pm}$, conversion fractions of DLX to 4-HD via the pre-systemic metabolism; $CL_{pm}$, clearance of DLX via the conversion to 4-HD via the systemic metabolism; $CL_{p}$, clearance of DLX via all elimination routes except the conversion to 4-HD via the systemic metabolism; $K_{a}$*,* $V_{p}$*,* and $F_{p}$: absorption rate constant, volume of distribution, and bioavailability of DLX after the pre-systemic metabolism, respectively; and $CL_{m}$*,* $V_{m}$*,* and $F_{m}$: clearance, volume of distribution, and bioavailability of 4-HD, respectively.

**Table 3:** Predicted PK parameters of DLX and 4-HD in humans extrapolated from rats and experimental human parameters in literatures.

Parameters	Unit	Predicted Value
$\;K_{a}$	1/h	0.687
$\;CL_{p}/F_{p}$	L/h	34.1
$\;CL_{pm}/F_{p}$	L/h	21.8
$\;V_{p}/F_{p}$	L	1022
$\;K_{ep}$	1/h	0.0547
$\;CL_{m}/F_{m}$	L/h	212
$\;CL_{m}/F_{m}$	L/h	5894

An average of human body weight of 70 kg was assumed. $K_{a}$ was predicted based on the relationship between $\;K_{a}$, $\;K_{e}$, and $T_{max}$ as: $T_{max}\;=\;\frac{\ln Ka-\ln Ke}{Ka-Ke}$, where $\;T_{max}$ = 4 h and $\;K_{e}\;=\;\frac{CLpm+CLp}{Vp}$.

**Table 4:** Pharmacokinetic (PK) parameters of DLX in humans according to different co-administered doses of PPL.

Scenario		Parameter	PPL 0 mg	PPL 5000 mg	Difference ^a^ (%)	PPL 15,000 mg	Difference ^b^ (%)
		$t_{1/2}$	12.7 ± 0.602	13.8 ± 0.699		15.3 ± 0.867	
Single dose	40 mg	$C_{max}$	12.9 ± 0.0928				
		$AUC_{0-t}$	294 ± 13.8				
	60 mg	$C_{max,SS}\;$	19.4 ± 0.139				
		$AUC_{0-t}$	441 ± 20.7				
Multiple dose, once daily	40 mg	$C_{max,SS}\;$	18.2 ± 0.557	19.7 ± 0.671	8.35	21.9 ± 0.868	20.2
		$AUC_{SS}$	294 ± 14.0	328 ± 16.8	11.6	376 ± 21.5	28.0
	60 mg	$C_{max,SS}\;$	27.3 ± 0.836	29.6 ± 1.01	8.35	32.8 ± 1.30	20.2
		$AUC_{SS}$	441 ± 21.0	492 ± 25.2	11.6	561 ± 32.3	28.0
Multiple dose, twice daily	40 mg	$C_{max,SS}\;$	28.7 ± 1.16	31.6 ± 1.39	10.2	35.7 ± 1.78	24.6
		$AUC_{SS}$	294 ± 14.0	328 ± 16.8	11.6	376 ± 21.5	28.0
	60 mg	$C_{max,SS}\;$	43.0 ± 1.74	47.4 ± 2.08	10.2	53.6 ± 2.67	24.6
		$AUC_{SS}$	441 ± 21.0	492 ± 25.2	11.6	564 ± 32.3	28.0

$C_{max}$ and $C_{max,SS}$, maximum concentration of DLX after the first dose and at steady state condition, respectively; $AUC_{0-t}$*_,_*area under the concentration-time curve from zero to last time after single DLX dose; $AUC_{SS}$ area under the concentration-time curve during one dosing interval at the steady state condition; the percentage difference in DLX PK parameters when drug was administered alone (PPL 0 mg) and (a) with PPL 5000 mg or (b) with PPL 15,000 mg, respectively; and unit: $t_{1/2}$ h; $C_{max}$ and $C_{max,SS}$ ng/mL; and $AUC_{0-t}$ and $AUC_{SS}$ h*ng/mL.

**Table 5:** Major ingredients and their respective contents in PPL extract.

Ingredient	Content (µg/mg) *		
Chrysin	23.57	±	3.02
Galangin	7.45	±	0.51
Kaempferide	4.30	±	0.57
Kaempferol	4.19	±	0.27
Caffeic acid phenethyl ester	0.35	±	0.01
Apigenin	0.19	±	0.10
Artepillin C	0.05	±	0.04
p-Coumaric acid	0.03	±	0.01
Caffeic acid	0.01	±	0.00

* Each value represents the mean ± standard deviation (*n* = 3 samples).

## Supplementary Materials

Supplementary Materials are available online at https://www.mdpi.com/1422-0067/21/5/1862/s1.
