# Supplementary material for: Application of an Inter-Species Extrapolation Method for the Prediction of Drug Interactions between Propolis and Duloxetine in Humans
Source: Int J Mol Sci. 2020 Mar 9;21(5):1862. doi: 10.3390/ijms21051862 (PMC7084906; doi:10.3390/ijms21051862)
Supplement: Supplementary file 1 [file ijms-21-01862-s001.pdf]

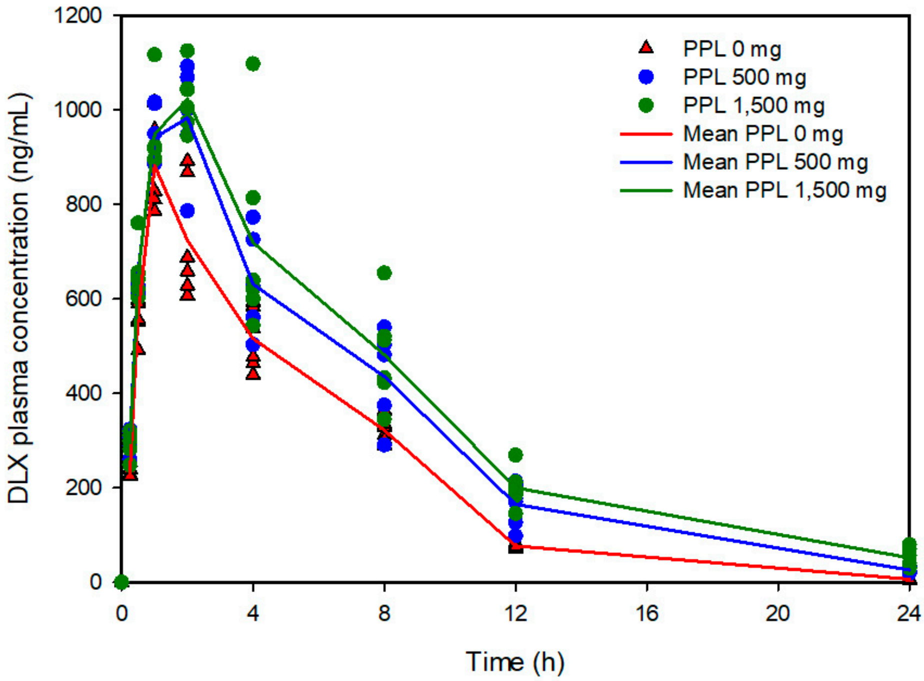

Figure S1A. Individual and mean of population of plasma concentration-time curve of DLX after oral administration of DLX in rats (n=6/group)

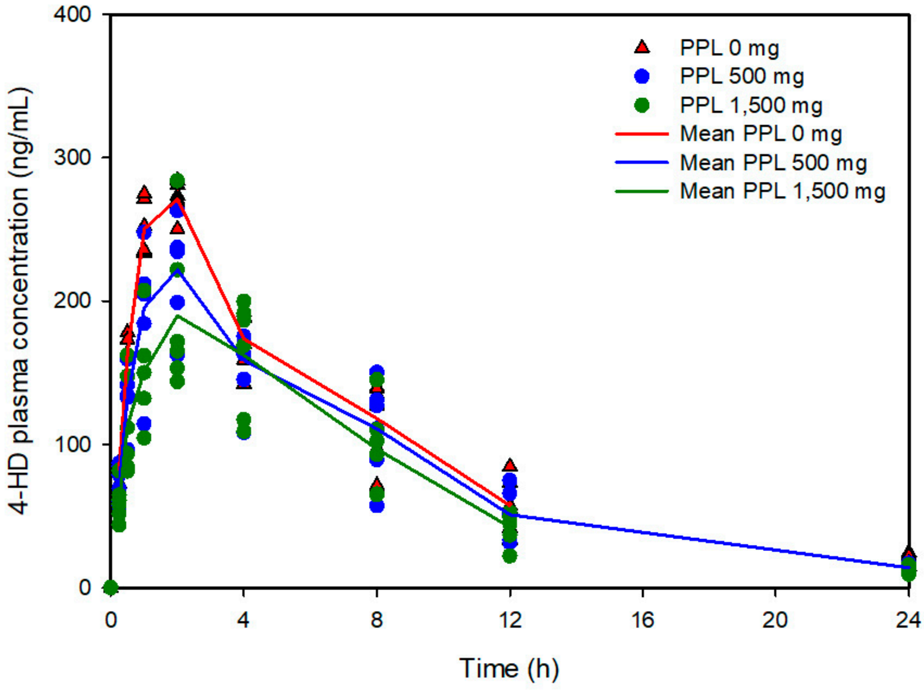

Figure S1B. Individual and mean of population of plasma concentration-time curve of 4-HD after oral administration of DLX in rats (n=6/group)
